# TRPV2 is a novel biomarker and therapeutic target in triple negative breast cancer

**DOI:** 10.18632/oncotarget.9663

**Published:** 2016-05-27

**Authors:** Mohamad Elbaz, Dinesh Ahirwar, Zhang Xiaoli, Xinyu Zhou, Maryam Lustberg, Mohd W. Nasser, Konstantin Shilo, Ramesh K. Ganju

**Affiliations:** ^1^ Department of Pathology, Wexner Medical Center, Ohio State University (OSU), Columbus, OH, USA; ^2^ The Comprehensive Cancer Center, Ohio State University (OSU), Wexner Medical Center, Columbus, OH, USA; ^3^ Center for Biostatistics, Ohio State University (OSU), Columbus, OH, USA; ^4^ Department of Internal Medicine, Ohio State University (OSU), Columbus, OH, USA; ^5^ Department of Pharmacology, Pharmacy School, Helwan University, Helwan, Egypt; ^6^ Department of surgery, Davis Heart and Lung Research Institute, Ohio State University, Columbus, OH, USA

**Keywords:** TRPV2, chemotherapy, TNBC, uptake, doxorubicin

## Abstract

Transient receptor potential vanilloid type-2 (TRPV2) is an ion channel that is triggered by agonists like cannabidiol (CBD). Triple negative breast cancer (TNBC) is an aggressive disease with limited therapeutic options. Chemotherapy is still the first line for the treatment of TNBC patients; however, TNBC usually gains rapid resistance and unresponsiveness to chemotherapeutic drugs. In this study, we found that TRPV2 protein is highly up-regulated in TNBC tissues compared to normal breast tissues. We also observed that TNBC and estrogen receptor alpha negative (ERβ-) patients with higher TRPV2 expression have significantly higher recurrence free survival compared to patients with lower TRPV2 expression especially those who were treated with chemotherapy. In addition, we showed that TRPV2 overexpression or activation by CBD significantly increased doxorubicin (DOX) uptake and apoptosis in TNBC cells. The induction of DOX uptake was abrogated by TRPV2 blocking or downregulation. *In vivo* mouse model studies showed that the TNBC tumors derived from CBD+DOX treated mice have significantly reduced weight and increased apoptosis compared to those treated with CBD or DOX alone. Overall, our studies for the first time revealed that TRPV2 might be a good prognostic marker for TNBC and ERβ- breast cancer patient especially for those who are treated with chemotherapy. In addition, TRPV2 activation could be a novel therapeutic strategy to enhance the uptake and efficacy of chemotherapy in TNBC patients.

## INTRODUCTION

Breast cancer is one of the most frequent causes of death among women in the United States [1]. Excluding non-melanoma skin cancers, breast cancer accounts for 23% of all cancers in women worldwide [1]. TNBC is one of the most aggressive breast cancer subtypes and is characterized by loss of estrogen receptor (ER), progesterone receptors (PR) as well as Human epidermal growth factor receptor-2 (Her2/neu) [2]. Therefore, TNBC is unresponsive to hormonal therapies of breast cancer such as ER/PR antagonists or trastuzumab therapies. In addition, the majority of TNBC patients develop rapid resistance to the standard TNBC chemotherapeutic drugs including taxanes, anthracyclins and cisplatin [3–7].

Although transient receptor potential (TRP) channels have been shown to play an important role in pain and temperature sensation, recent studies suggest they may also regulate tumor growth and metastasis [8]. TRP channels are proteins consisting of six trans-membrane segments [9]. There are six families of TRP channels which show similarities in sequence homology and permeability to cations. However, each family responds differently to different external stimuli and local environment [9]. These channels are considered as molecular sensors that play different roles in physiological and pathological conditions [9].

Transient Receptor Potential Vanilloid type 2 (TRPV2) is a member of TRPV family that responds to noxious heat, cell membrane stretch and osmolarity changes [10]. The TRPV2 channel is activated by agonists such as Δ9-tetrahydrocannabinol (Δ9-THC) and cannabidiol (CBD) [11, 12]. TRPV2 has also been reported to be induced by growth factors which leads to PI-3K-dependent and independent TRPV2 translocation towards the plasma membrane [13, 14]. CBD has been shown to exert anti-proliferative and anti-metastatic activities against breast cancer cells [15, 16].

Previous studies showed that ion channels such as TRPV4 enhanced cell’s uptake ability to aminoglycoside antibiotics and the mutation of the functional pore region abrogates this uptake [17]. However, not much is known about the role of TRPV2 in breast cancer and drug uptake. In the present study, we analyzed TRPV2 expression levels in TNBC and normal breast tissues. We also analyzed the importance of TRPV2 as a prognostic marker for TNBC patients. Furthermore, we determined the efficacy of TRPV2 activation in enhancing the anti-tumor activity of chemotherapeutic drugs and how it can affect its apoptotic potential *in vitro* and *in vivo*.

## RESULTS

### TRPV2 is highly expressed in TNBC tissues and associated with better prognosis

**Figure 1:**
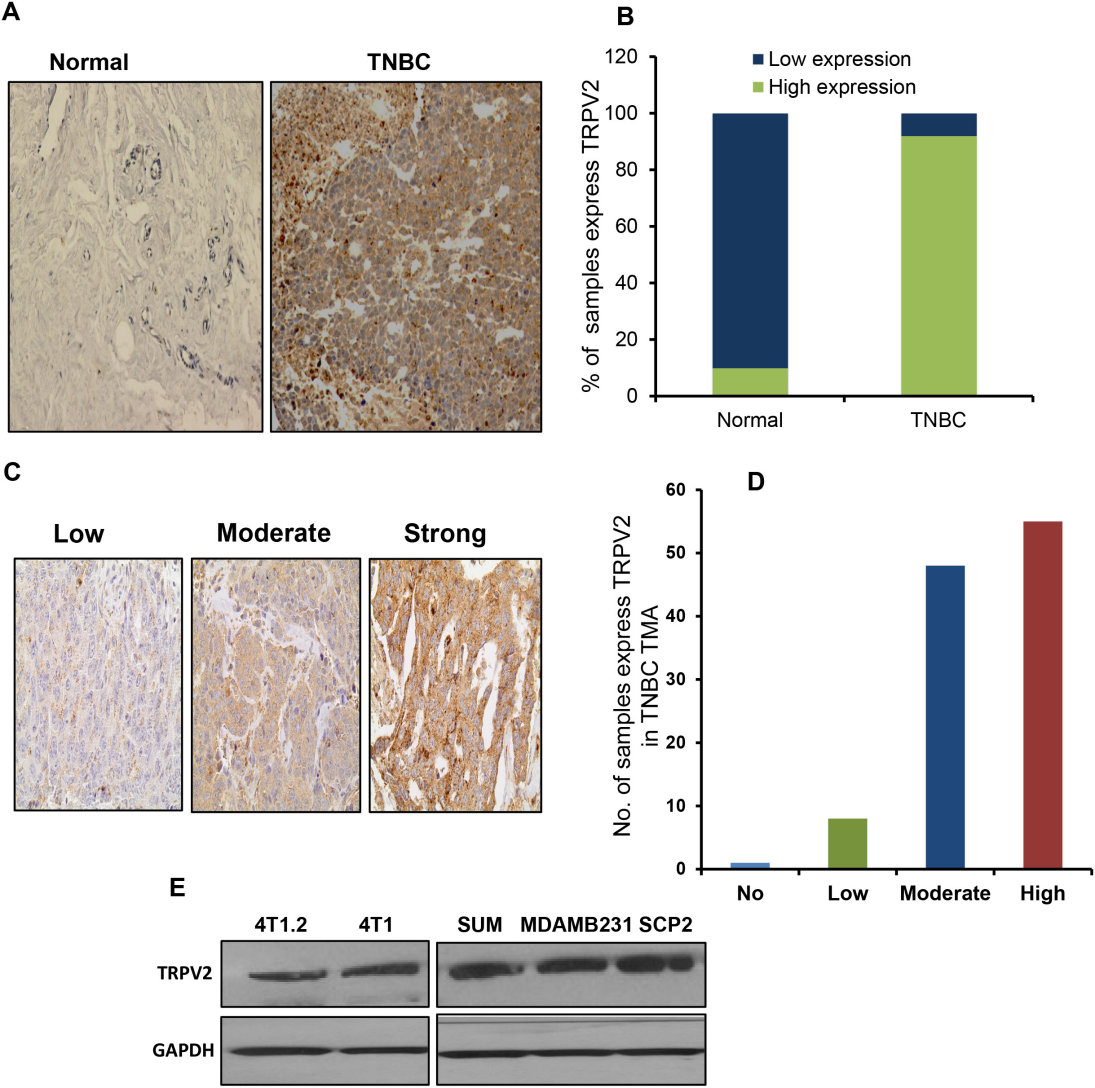

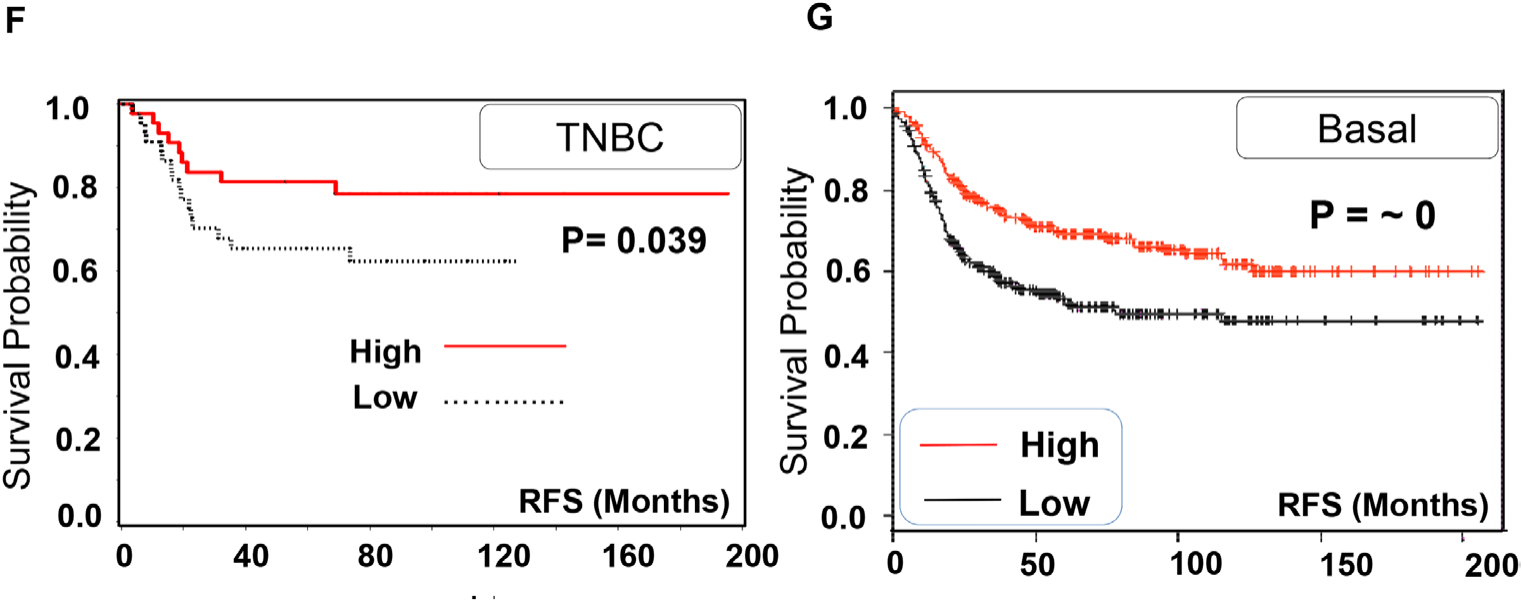
TRPV2 is highly expressed in TNBC tissues and associated with better prognosis **A.** Representative image of immune-histochemical (IHC) staining showing TRPV2 expression in normal and TNBC breast tissues. **B.** Quantitation of TRPV2 expression of normal and TNBC breast tissues. **C.** Representative image of IHC staining showing different grades of TRPV2 expression in the tissue microarray (TMA) of TNBC patient samples that has been used in the study. **D.** Quantitation of TRPV2 expression in the tissue microarray (TMA) of TNBC patient samples that has been used in the study. **E.** Western blot image showing TRPV2 protein expression of 4T1.2, 4T1, SUM159, MDA-MB231 and SCP2 breast cancer cell lines. GAPDH has been used as a loading control. **F.** Tissue microarray analysis showing the recurrence free survival (RFS) of TNBC patients (n=116) of high/low TRPV2 protein expression.G. Kaplan Meier blot showing RFS of high/ low expressing TRPV2 breast cancer patients of basal subtypes (n= 580 patients). P values are provided for each Kaplan Meier graph.

We examined the expression pattern of TRPV2 in normal breast tissues versus TNBC tissues. We found that TRPV2 is significantly highly expressed in TNBC tissues compared to normal breast tissues (P< 0.001) (Figure 1A-1B). Next, we analyzed TRPV2 expression in a tissue microarray (TMA) that have human TNBC tissue samples (number of patients = 116) and we observed strong or moderate expression of TRPV2 in most of the TNBC tissues (∼90%) (Figure 1C-1D). We also observed that TNBC cell lines express TRPV2 protein (Figure 1E). Furthermore, we found that TNBC patients, who express higher level of TRPV2 protein, have significantly higher recurrence free survival (RFS) than patients with lower TRPV2 protein expression (Figure 1F). We confirmed these results by analyzing the correlation of TRPV2 expression in basal breast cancer subtype on RFS using publically available Kaplan Meier plotter [18]. As shown in Figure 1G, patients with higher level of TRPV2 have significantly higher RFS than the patients with lower TRPV2 expression level. These results indicate that TRPV2 is highly expressed in TNBC tissues compared to normal breast tissues. In addition, higher TRPV2 expression is correlated with better prognosis in TNBC/Basal subtype.

### TRPV2 expression is associated with better prognosis of ERα- patients especially those who receive chemotherapy

**Figure 2:**
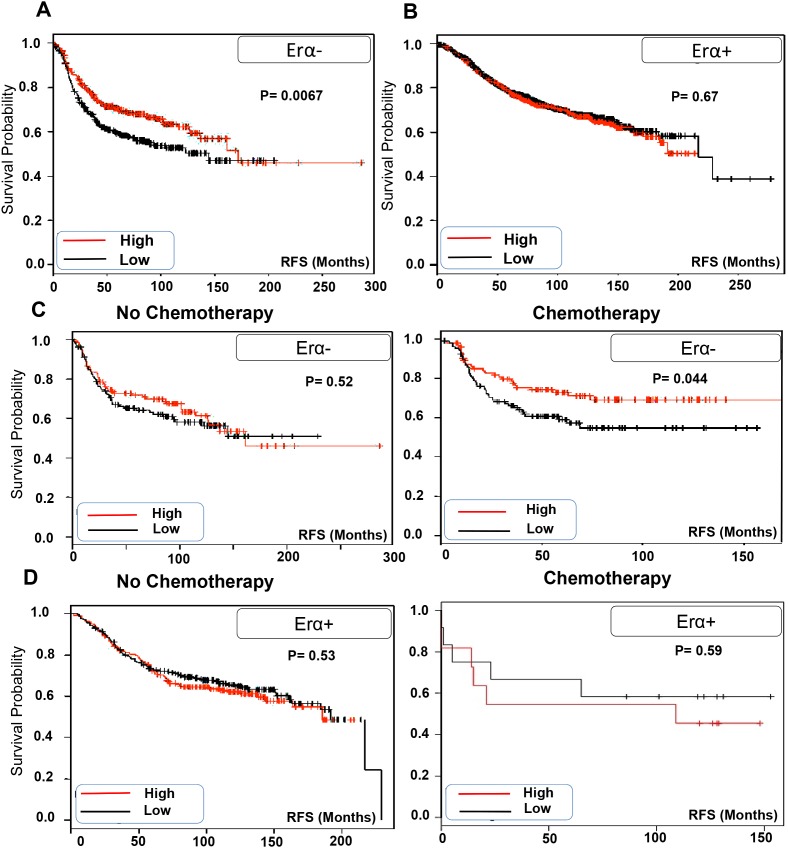
TRPV2 expression is correlated with better prognosis of ERα- patients especially those who receive chemotherapy **A.** Kaplan Meier blot showing recurrence free survival (RFS) of high/low expressing TRPV2 ERα- breast cancer patients (n= 671). **B.** Kaplan Meier blot showing recurrence free survival (RFS) of high/low expressing TRPV2 ERα+ breast cancer patients (n= 1802). P values are provided for each Kaplan Meier graph. **C.** Kaplan Meier blot showing recurrence survival of high/low expressing TRPV2 ERα- breast cancer patients who received (n=211) or not chemotherapy (n=253) medication. **D.** Kaplan Meier blot showing recurrence survival of high/low expressing TRPV2 ERα+ breast cancer patients who received (n=500) or not chemotherapy (n=23) medication.

We analyzed the correlation of TRPV2 expression with the clinical outcome of ERα- breast cancer patients using publically available Kaplan Meier plotter [18]. We found that ERα- breast cancer patients who express higher level of TRPV2 have significantly higher RFS than patients with lower TRPV2 expression level (Figure 2A). In contrast, TRPV2 expression level does not affect RFS in ERα+ breast cancer patients (Figure 2B). Next, we analyzed the correlation of TRPV2 expression to the prognosis of chemotherapy-treated breast cancer patients using Kaplan Meier datasets. As shown in Figure 2C, ERα- breast cancer patients who have higher TRPV2 expression and receive chemotherapy treatment show better RFS than those who have lower expression of TRPV2. In contrast, no significant difference in prognosis was observed between non-chemotherapy treated ERα- breast cancer patients who have either high or low TRPV2 expression. In case of ERα+ breast cancer patients, TRPV2 expression level does not affect RFS whether the patients receive chemotherapy or not (Figure 2D). These results suggest that higher TRPV2 expression might be considered as a good prognostic marker for ERα- patients especially those who are receiving chemotherapy in their treatment plan.

### Cannabidiol enhances the efficacy and the apoptotic potential of chemotherapeutic drugs

**Figure 3:**
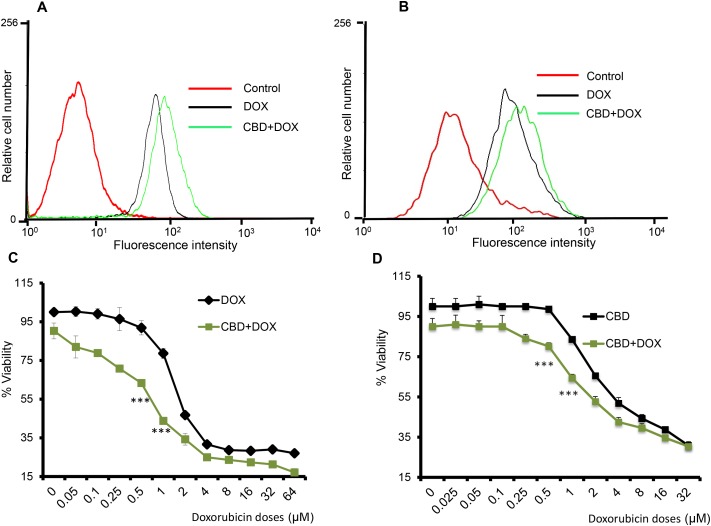

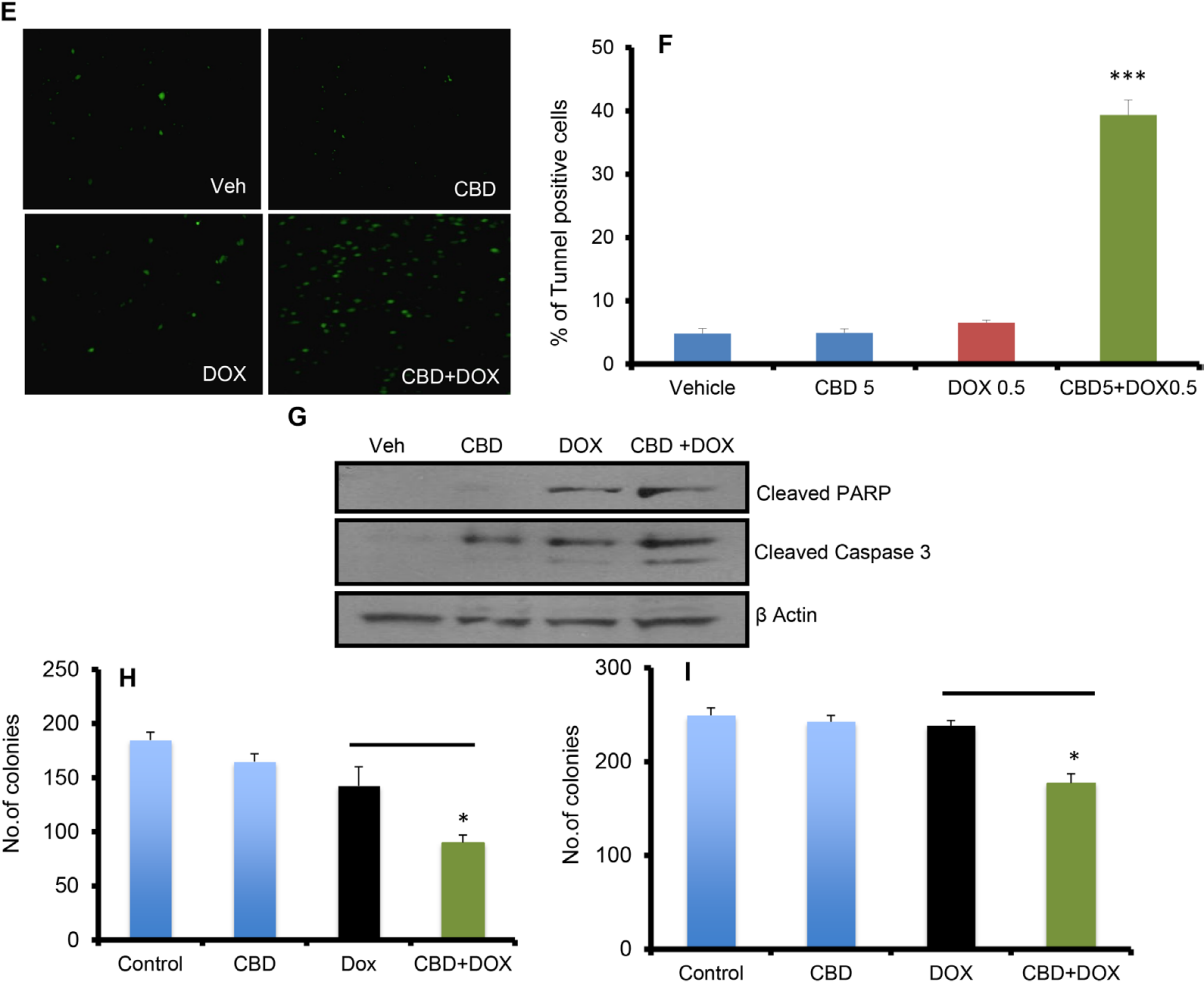
CBD potentiates the apoptotic effect of chemotherapeutic agents **A.** SUM159 cells were treated with Vehicle or CBD for 2h followed by DOX for 30 min then subjected to FACS analysis of DOX uptake. **B.** MDA-MB231 cells were treated with Vehicle or CBD for 2h followed by DOX for 30 min then subjected to FACS analysis of DOX uptake. SUM159 **C.** or MDA-MB231 **D.** cells were treated with different concentrations of DOX in presence or absence of CBD for 24 hours and subjected to MTT assay. **E.** SUM159 cells were treated with CBD 5μM or DOX 0.5μM or combination of CBD and DOX and subjected to tunnel assay. **F.** Quantification of the % of tunnel positive cells has been calculated. **G.** SUM159 cells were treated with Veh or CBD 5μM or DOX 0.5μM or CBD+DOX for 24 h and the lysates were subjected to western blot analysis to detect the indicated proteins. SUM159 **H.** or MDA-MB231 **I.** cells were treated with 0.5μM DOX in presence or absence of CBD (5μM) for 6 days in reduced serum media and the number of formed colonies has been counted.

Since we observed better prognosis in chemotherapy treated patients that had higher TRPV2 expression, we analyzed whether TRPV2 activation would affect chemotherapeutic drug uptake and efficacy. TRP channels have been previously reported to participate in selective uptake of xenobiotics into target cells [17, 19]. Doxorubicin (DOX) is an anthracycline chemotherapeutic drug with a small molecular weight and it has natural fluorescent properties [20] that confers an excellent option to study the chemotherapeutic drug uptake by flowcytometry. Cannabidiol (CBD) has been shown to act as a TRPV2 agonist. To study the potential of TRPV2 activation in enhancing the chemotherapeutic drug uptake in TNBC, we treated SUM159 and MDA-MB231 cells with CBD (5 μM) for two hours followed by DOX (5 μM) for 30 minutes and we checked for DOX-positive cells by flowcytometric analysis. As shown in Figure 3A-3B, TNBC cells that were treated with CBD in combination with DOX have higher uptake of DOX compared to cells treated with DOX only.

Next, we evaluated whether this increase of DOX uptake is associated with a decrease in TNBC cell viability and an increase in apoptosis. As shown in Figure 3C-3D, SUM159 and MDA-MB231 cells that were treated with DOX in presence of CBD showed reduced viability compared to DOX alone treated cells. Similarly, we found that CBD enhanced paclitaxel efficacy in SUM159 cells (Supplementary Figure 2). We also observed that cells that were treated with CBD (5 μM) in combination with DOX had significantly higher apoptosis signals compared to the cells treated with CBD or DOX alone as determined by tunnel assay (Figure 3E-3F). Next, we analyzed the effect of CBD and DOX treatments on some apoptotic markers such as cleaved poly ADP ribose polymerase (PARP) and cleaved caspase-3. We found that CBD upregulated the levels of cleaved caspase-3 and cleaved PARP when co-administrated with DOX compared to CBD or DOX single treatments (Figure 3G and Supplementary Figure 1A-1B).

In order to test the ability of the combination therapy to inhibit cancer cell’s ability to survive, proliferate and form single cell clones, we performed colony-forming assay. We found that CBD in combination with DOX significantly inhibited the number of SUM159 and MDA-MB231 colonies compared to CBD or DOX alone (Figure 3H-3I). These results show that TRPV2 activation increases the uptake of chemotherapeutic agents, which in turn is associated with higher apoptotic potential and higher efficacy of chemotherapeutic drugs against TNBC cells.

### Overexpression of TRPV2 increased doxorubicin uptake and efficacy

**Figure 4:**
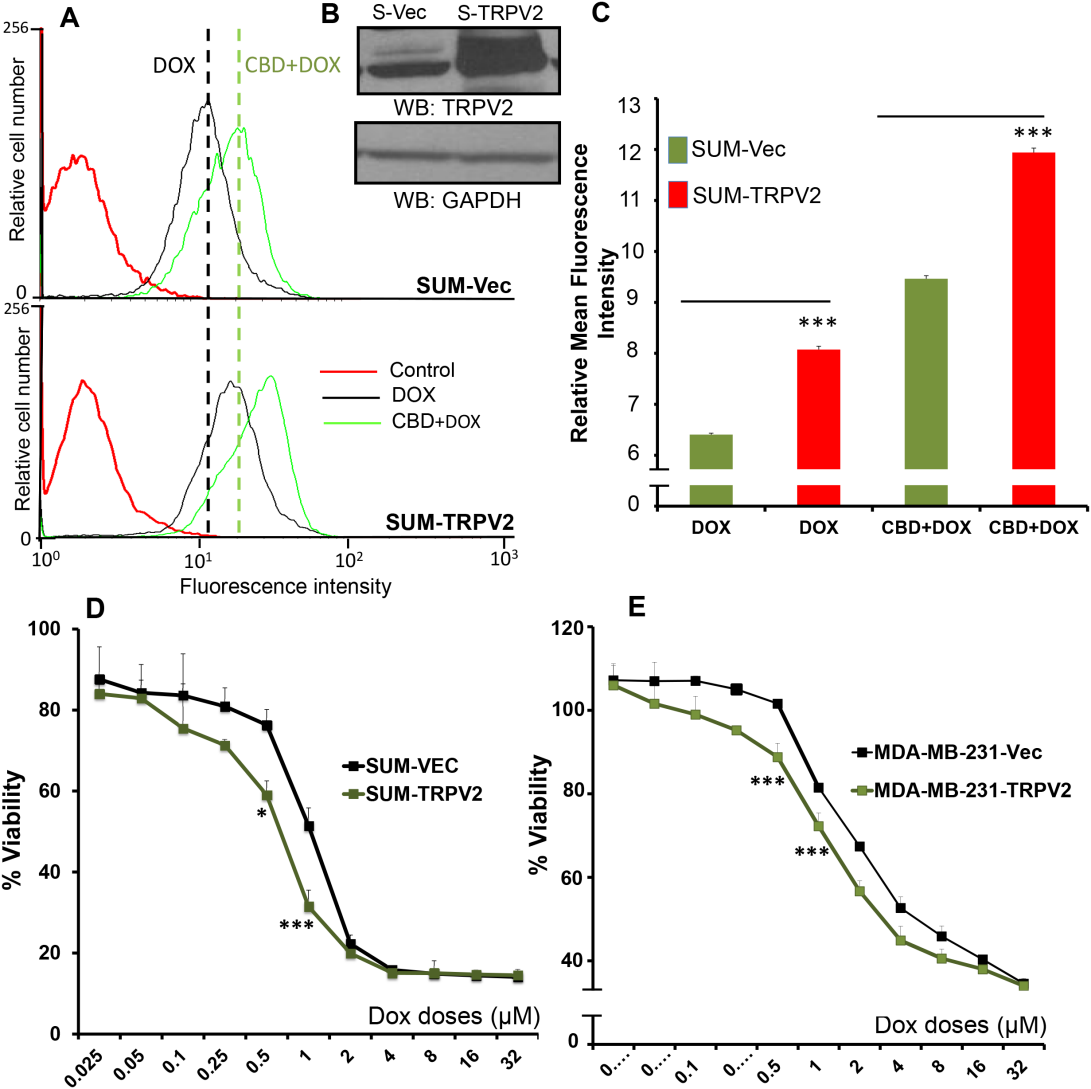
TRPV2 Overexpression enhances uptake of doxorubicin in SUM159 cells **A.** SUM159 cells were transfected with empty vector (SUM-Vec) or with full-length TRPV2 plasmid (SUM-TRPV2) and these cells were treated 0.5μM DOX for 30 min with or without CBD for 2 hours. Cells were collected for FACS analysis to detect DOX positive cells. **B.** Western blot image showing TRPV2 expression in empty vector transfected SUM159 cells (S-Vec) and TRPV2 overexpressing SUM159 cells (S-TRPV2). **C.** Quantification of FACS data for DOX uptake in SUM-Vec and SUM-TRPV2 cells. **D.** SUM-Vec or SUM-TRPV2 cells were treated with different concentration of DOX for 24 hours and subjected to MTT assay. **E.** MDA-MB231 cells were transfected with empty vector (MDA-MB231-Vec) or with full-length TRPV2 plasmid (MDA-MB231-TRPV2) cells and then were treated with different concentration of DOX for 24 hours and subjected to MTT assay.

Next we confirmed the role of TRPV2 in enhancing DOX-mediated effects by overexpressing TRPV2 in TNBC breast cancer cell line SUM159 and analyzed the drug uptake potential of these cells. As shown, TRPV2 overexpressing cells showed higher uptake of DOX before and after CBD treatment, compared to empty vector-transfected cells (Figure 4A-C and Supplementary Figure 1C).

We also analyzed the effect of TRPV2 overexpression on DOX-mediated viability. We found that TRPV2 overexpressing cells (SUM159 and MDA-MB231) showed reduced viability after DOX treatment compared to empty vector-transfected cells (Figure 4D-4E). These results suggest that the uptake of the chemotherapeutic drugs is triggered, in part, by the TRPV2 channel. Furthermore, higher expression level of TRPV2 is associated with higher efficacy of chemotherapeutic agents.

### TRPV2 blocking, downregulation or interference abrogates CBD-mediated DOX uptake and apoptotic activation

**Figure 5:**
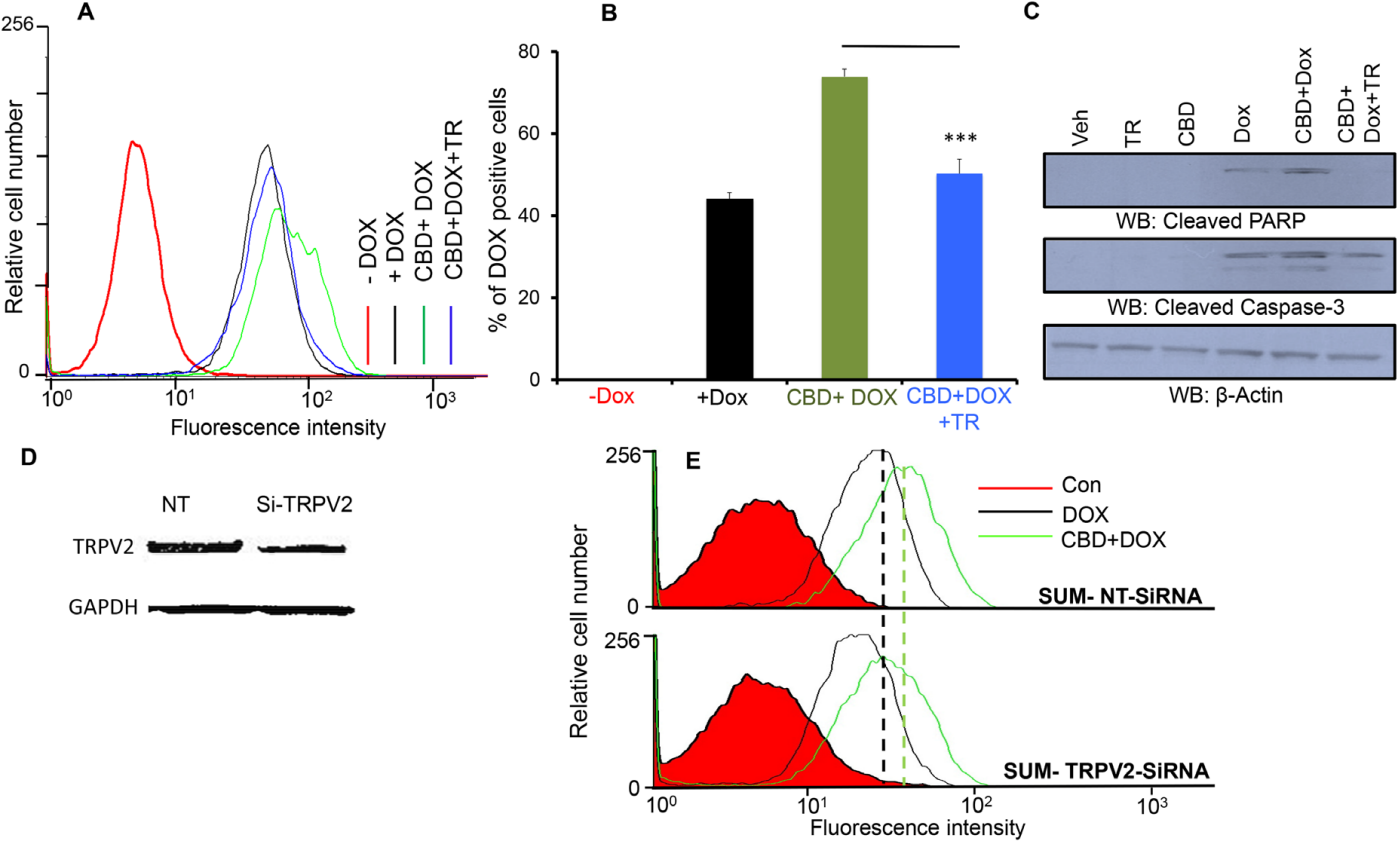

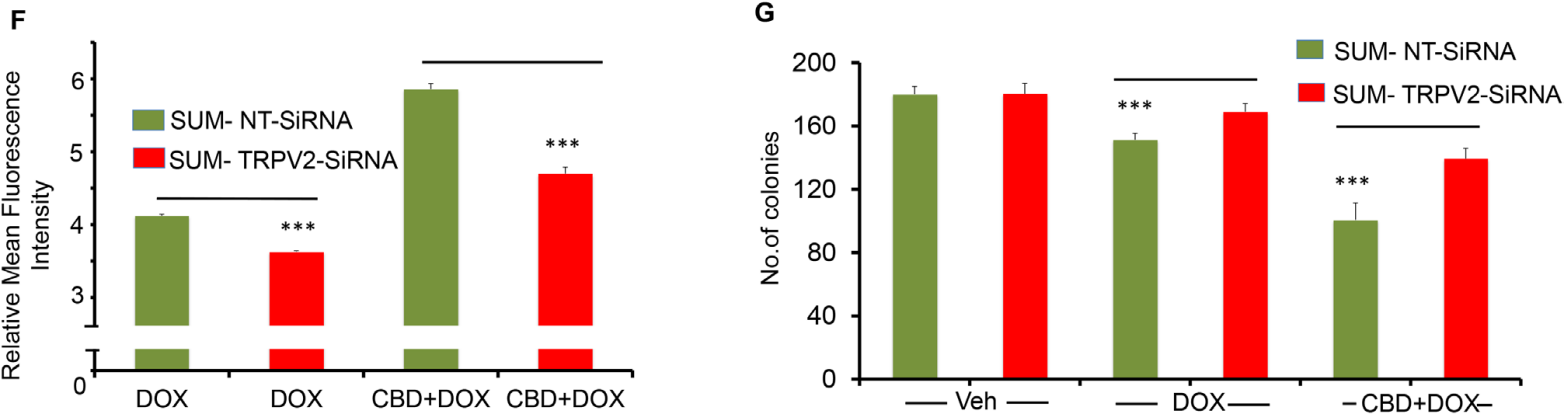
TRPV2 inhibition suppresses CBD and DOX-mediated cytotoxic effects **A.** SUM159 cells were treated with Vehicle or CBD or Tranilast (TR) or CBD + Tranilast for 2h followed by DOX for 30 min then subjected to FACS analysis of DOX uptake. **B.** Quantitation of the % of DOX-positive cells of the uptake experiments of the four groups. **C.** SUM159 cells were treated with the indicated treatments and the lysates were collected and immunoblotted against the indicated proteins. **D.** Western blot image showing TRPV2 expression in non-targeting small interfering RNA (SUM-NT SiRNA) and TRPV2-targeting small interfering RNA (SUM-TRPV2-SiRNA) transiently transfected cells. **E.** SUM-NT SiRNA or SUM-TRPV2-SiRNA transfected cells were treated with 0.5 μM DOX for 30 min in the presence or absence of CBD for 2 hours. Cells were collected for FACS analysis. **F.** Quantification of the mean florescence intensity (MFI) has been presented. **G.** SUM-NT SiRNA or SUM-TRPV2-SiRNA transfected cells have been used for colony formation assay in the presence or absence of DOX and/or CBD (5μM) and the number of colonies have been counted.

In order to validate whether the CBD’s effects are mediated through TRPV2, we used a TRPV2 pore blocker (Tranilast) [21–23]. As shown, TRPV2 blocking abrogated DOX uptake that has been increased by CBD treatment (Figure 5A-5B). On the molecular level, we found that the TRPV2 pore region blocker reduced the expression levels of the cleaved PARP and the cleaved caspase-3 proteins back to their basal levels (Figure 5C, Supplementary Figure 1D-1E). We have also downregulated TRPV2 by SiRNA (200 nM) transfection (Figure 5D, Supplementary Figure 1F). TRPV2 downregulation inhibited DOX uptake and it also abrogated DOX and CBD effects on reducing the number of SUM159 colonies (Figure 5E-G). This data suggest that TRPV2 plays an important role in regulating chemotherapeutic drug uptake and efficacy. These results also confirm that TRPV2 activation is responsible for CBD-mediated effects on chemotherapeutic uptake and subsequent apoptotic activities.

### CBD improved the anti-tumor chemotherapeutic efficacy *in vivo*

**Figure 6:**
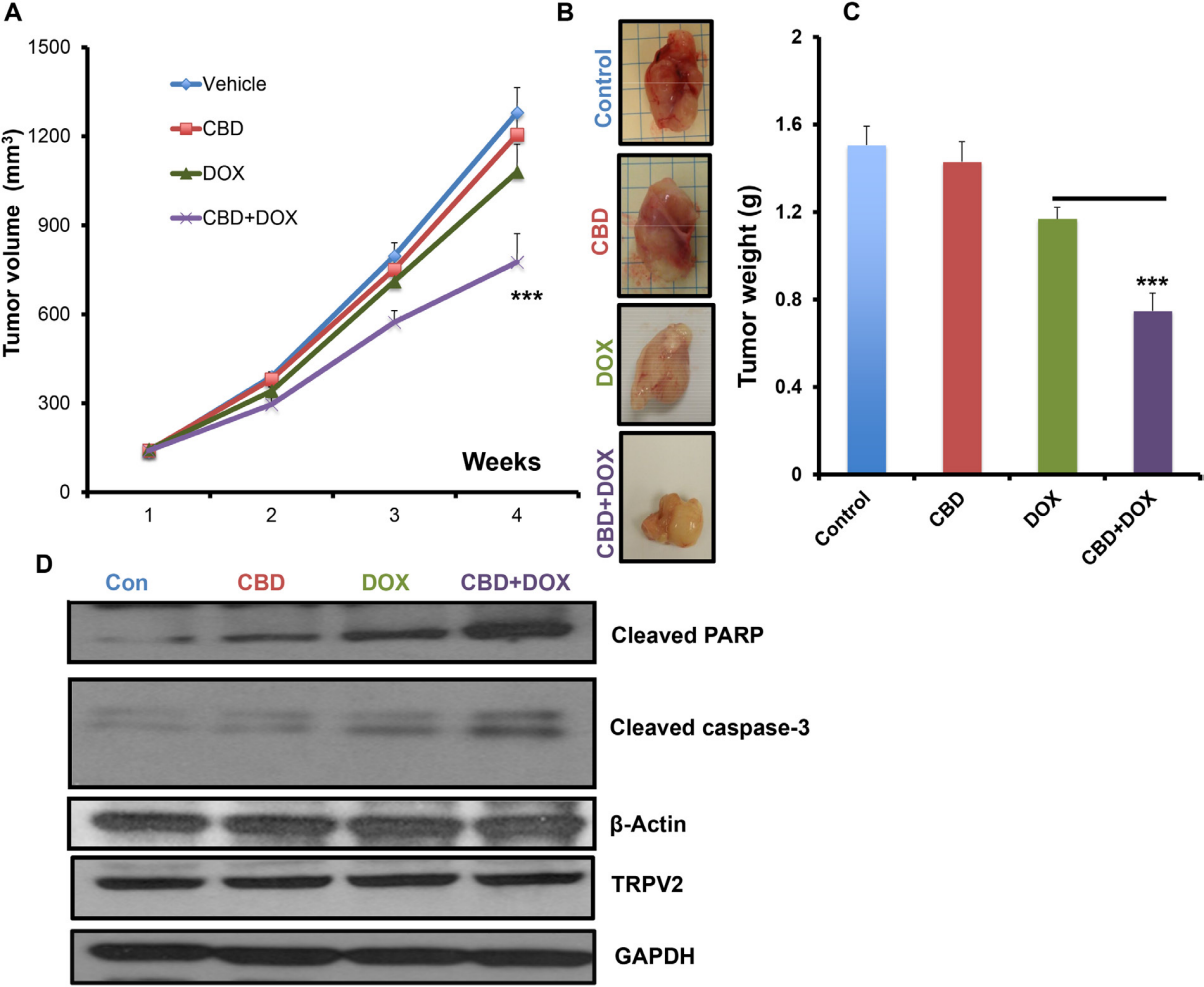
CBD improved the anti-tumor chemotherapeutic efficacy *in vivo* Nude mice were orthotopically injected into 4th mammary gland with SUM159 cells and subjected to the indicated treatments for 4 weeks and the tumor volume **A.** has been measured every week and the weight **B.** of the dissected tumors has been determined for each group. **C.** Representative images of dissected tumors from the indicated experimental groups. **D.** Tumor lysates from the experimental groups were used for western blot analysis and immunoblotted against the indicated proteins.

To examine the ability of CBD in increasing the chemotherapeutic drug efficacy *in vivo*, we used a Nude mice model injected orthotopically with SUM159 cells in 4^th^ mammary gland. The mice were treated with either vehicle, CBD, DOX or CBD in combination with DOX. As shown, the CBD+DOX group showed significantly lower tumor volume and weight compared to single treatments of CBD or DOX alone (Figure 6A-C). Then, we used the tumor lysates for the analysis of some apoptotic markers. As shown, CBD+DOX treated tumors have higher levels of cleaved caspase-3 and cleaved PARP compared to the single treatment groups (Figure 6D, Supplementary Figure 1G-I). These results suggest that CBD could enhance the chemotherapeutic efficacy *in vivo* and inhibit the tumor growth of breast cancer cells.

## DISCUSSION

TNBC is a very aggressive subtype of breast cancer due to its lack of the hormonal receptors as well as HER-2 and thus no targeted therapy is available. Till now, chemotherapy is the first choice to treat TNBC, however, resistance, relapse, poor response rate and toxicity are common problems associated with chemotherapeutic drugs [24]. Thus, there is an urgent need for developing new strategies to increase the efficacy and reduce the toxicity of the chemotherapeutic drugs. One of these strategies is to enhance the sensitivity of TNBC cells towards the chemotherapeutic drugs. In this study, we showed that TRPV2 protein is highly expressed in TNBC tissues compared to normal breast tissues. In addition, higher TRPV2 expression is associated with better prognosis in TNBC/basal and ERα- breast cancer patients especially those who were treated with chemotherapy. Finally, we showed that TRPV2 overexpression or activation enhances chemotherapy’s uptake and efficacy *in vitro* and *in vivo*.

TRPV2 expression has been studied in prostate, bladder cancer and hepatocellular carcinoma [25–27]. However, not much is known about its expression and role in breast cancer especially TNBC. In this study, we observed higher TRPV2 protein expression in TNBC compared to normal breast tissues. We also observed that higher TRPV2 expression is associated with better prognosis in TNBC/basal subtype patients. To our knowledge this is the first report showing TRPV2 expression in TNBC. There are few reports about its expression in breast cancer [28, 29]. We also found that high TRPV2 expression level is a good prognostic factor (better RFS) for ERα- and TNBC patients especially those who were treated with chemotherapeutic drugs. However, we have not found significant correlation between TRPV2 expression and RFS in ERα+ breast cancer patients. There are some reports that suggest a correlation between progesterone treatment and TRPC5 channel activity [30]. However, cross-talk between TRPV2 and estrogen receptor needs more investigation.

We also found that TRPV2 overexpression or its activation by CBD enhances the ability of TNBC cells to uptake DOX and this significantly enhances DOX anti-tumorigenic efficacy. In addition, we found that TRPV2 downregulation or blocking significantly inhibits TNBC cells’ ability to uptake DOX. Our study is in accordance to previous study that showed TRPV2 enhanced chemotherapeutic drug uptake in glioblastoma cell line [19].

Combination treatment of CBD and DOX against TNBC cells inhibited tumor volume, weight, and induced higher levels of cleaved PARP and cleaved caspase-3, compared to single treatments *in vitro* and *in vivo*. Nabissi et al; have shown that TRPV2 negatively controls cancer cell proliferation and inhibits their resistance to Fas-induced apoptosis [31]. Furthermore, Yamada et al, have reported that TRPV2 induces apoptotic cell death in bladder cancer cells [27]. A recent study also showed that TRPV2 promotes H2O2-induced oxidative stress and cytotoxicity in human hepatoma cells [32]. Our study shows that TRPV2 activation enhances apoptosis by increasing the uptake of pro-apoptotic agents. Combination of CBD with DOX *in vivo* showed significantly higher activity than DOX alone and no obvious signs of toxicity were observed in mice treated with combination treatment. This suggests that TRPV2 could be used as a target to achieve better precision medicine strategy. Based on our data, we expect that TNBC patients especially those who have higher TRPV2 expression could get the maximum benefit of chemotherapy alone or in combination with TRPV2 agonist (CBD).

In summary, our studies show that TRPV2 could be used as a novel biomarker for TNBC and basal-type breast cancer patients. TRPV2 expression also correlates with better RFS in chemotherapy-treated TNBC and ERα-patients. Furthermore, TRPV2 up-regulation and activation enhances the sensitivity of TNBC cells towards the chemotherapeutic drugs *in vitro* and *in vivo*. Overall, our study revealed that TRPV2 might be a good prognostic marker for TNBC patients and TRPV2 agonist (CBD) could be used as an adjuvant therapy to enhance the therapeutic response especially for TNBC patients who receive chemotherapy.

## MATERIALS AND METHODS

### Reagents and antibodies

The following reagents were purchased from different sources: Cannabidiol (Cayman), Doxurubicin HCL (Sigma Aldrich, Cat. # D1515), Paclitaxel (Taxol) (Sigma Aldrich, Cat. # T7402) and Tranilast. (Cayman Cat. # 13044). The following antibodies were purchased from different sources: Cleaved PARP (Cell Signaling Cat. # 5625P), cleaved caspase-3 (Cell Signaling Cat. # 9664P); GAPDH (Santa Cruz Cat. # FL-335) β-actin antibodies (Santa Cruz Cat. # H-300), and TRPV2 antibody (Sigma Cat. # HPA044993 and Abcam Cat. # ab6183).

### Cell culture

TNBC human breast cancer cell line (SUM159PT) [33] was obtained from Dr. Sarmila Majumder, The Ohio State University in 2013. The identity of the cells was regularly verified monthly based on their cell morphology. Cells were cultured in DMEM (Corning cellgro Cat.# MT 101-013-CV) containing 10% heat-inactivated fetal bovine serum (FBS) (Sigma Cat.# F0926), 5-units/mL penicillin, and 5 mg/mL streptomycin [34].

### Western blot analysis

Cells were plated and lysed in lysis buffer (RIPA). Tumor samples were processed and lysed for further analysis. Equal concentrations of total proteins were loaded on 4-12% SDS–polyacrylamide gels (NuPAGE Novex Cat.# NP0335BOX) and transferred to nitrocellulose membranes (BioRad) and blocked for 1 h with 5% milk. Membranes were then incubated with primary antibody overnight, then incubated for 1 h at RT with secondary antibodies. The membranes were then stained and developed using a chemiluminescence system (pierce ECL Cat.# 32106) and exposed to X-ray film (BioExpress F-9023-5X7).

### Immunohistochemistry

Antibody against TRPV2 (1:100, Sigma Aldrich) was applied to formalin fixed paraffin embedded tissues. Four micron sections were incubated with the primary antibody overnight at 4°C. Vectastain Elite ABC reagents with avidin DH:biotinylated horseradish peroxidase H complex with 3,3′-diaminobenzidine (Polysciences) and Mayer hematoxylin (Fisher Scientific) have been used for detection of the bound primary antibodies. Tumors samples were fixed, and embedded in paraffin. We performed tissue microarrays (TMA) staining to stain normal (n=10) and TNBC patient breast tissues (n=116 patients who were treated with multi-agent chemotherapy. TNBC patients’ recurrence free survival (RFS) has been documented at OSU medical center and analyzed after staining with Anti-TRPV2 antibody).

### Mouse model

All experimental procedures were approved by University Laboratory Animal Resources (ULAR) at The Ohio State University. Female NU/NU nude mice were purchased from (Charles River Laboratories Inc.). Tumors were induced by orthotopic injection of tumor cells SUM159 (5 x 10^6^) in 4^th^ mammary glands [35, 36]. When the tumors became palpable, mice were randomized, and injected once per week for 4 weeks with CBD (5 mg/kg) or vehicle peri-tumorally, then with doxorubicin (Dox) 5mg/kg I.P after two hours of CBD treatment.

### Plasmid constructs

Full length TRPV2 overexpressing plasmid has been purchased from (Origene). In addition, small interfering RNA (SiRNA) either non-targeting or TRPV2 Si-RNA have been purchased from (Dharmacon) company. Transfections have been done using Lipofectamine 2000 (Cat#11668-030) in reduced serum media according to the manufacturer’s recommendations. After 48 hours the cells were collected for protein expression analysis or used for subsequent experiments.

### Doxorubicin uptake

Cells are treated with CBD 5 uM for 2 hours followed by DOX (5 μM) for 30 minutes. Cells are then trypsinized and washed and analyzed using a BD FACS caliber, then plotted for analysis.

### Apoptosis assay

Detection of apoptosis in cells was performed using APO green TUNEL assay kit (Bio-ToolCat#: B31112). Cells were fixed, permeabilized and treated 1h with recombinant TdT enzyme and APO-Green labeling Mix. After that, cells were detected using fluorescent microscope (Olympus).

### Cell viability assay

10,000 cells were seeded in 96 well plates for 24 h and then treated with different conc. of DOX or Paclitaxel in presence of vehicle or CBD 5 μM for 24h in SFM and subjected to MTT assay (Roche Cat #: 11465007001) according to manufacturer’s protocol [37].

### Colony forming assay

Colony forming assay has been performed as described earlier [37].

### Statistical analysis

Results were represented as mean ± SD. Two-sample t-tests were used to compare vehicle and treated groups for each experiment with independent observations. A linear mixed effects model was used for the tumor volume data analysis to take account of the correlation among observations from the same animal, as the tumor volume was measured over time for each animal. For tumor weight and tumor volume analysis, ANOVA test has been used. Log rank tests were used to test whether TRPV2 expression measured with TMA is associated with recurrence free survival (RFS), and the Kaplan-Meier survival curves were used to display the results. P<0.05 was considered to be statistically significant. For all graphs, ^*^ indicates *P*<0.05, ^**^ indicates *P*<0.01 and ^***^ indicates *P*<0.001.

## SUPPLEMENTARY MATERIALS FIGURES



## Abbreviations

(TRPV2): Transient Receptor Potential Vanilloid type 2
(TNBC): Triple negative Breast cancer
(Dox): Doxorubicin
(cannabidiol): CBD
