# Supplementary material for: TRPV2 is a novel biomarker and therapeutic target in triple negative breast cancer
Source: Oncotarget. 2016 May 27;9(71):33459–70. doi: 10.18632/oncotarget.9663 (PMC6173360; doi:10.18632/oncotarget.9663)
Supplement: Supplementary file 1 [file oncotarget-09-33459-s001.pdf]

TRPV2 is a novel biomarker and therapeutic target in triple negative breast cancer

SUPPLEMENTARY FIGURES

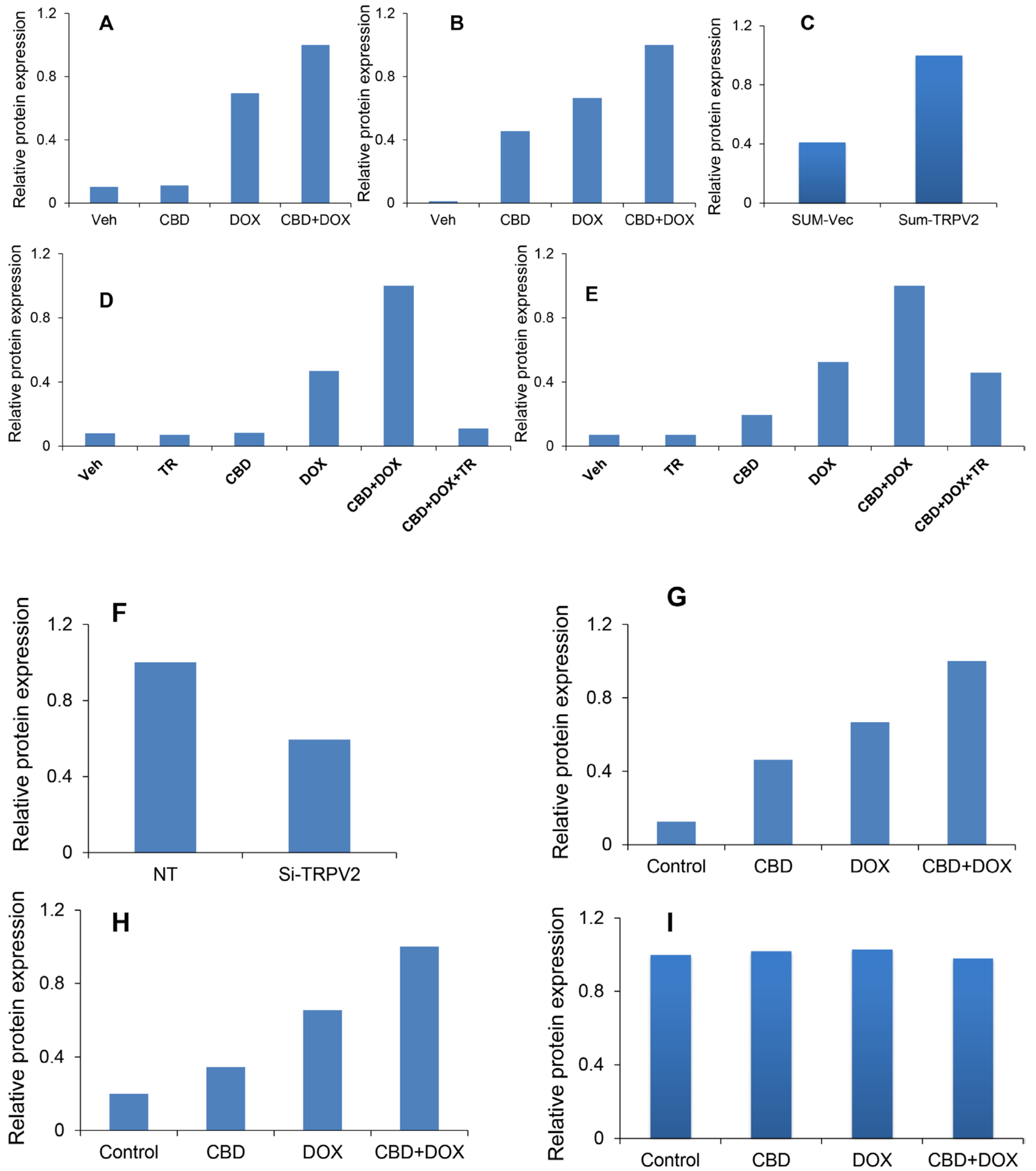

Supplementary Figure S1: Quantification of protein expression. Protein expressions in figure 3-G have been quantified as a ratio between the protein of interest to the loading control as follow **A.** Cleaved PARP/ β-Actin **B.** Cleaved Caspase-3/ β-Actin. **C.** Protein expression in figure 4-B has been quantified as a ratio between TRPV2 to GAPDH in empty vector transfected SUM159 cells (SUM-Vec) and TRPV2 overexpressing SUM159 cells (SUM-TRPV2). Protein expressions in figure 5-C have been quantified as a ratio between the protein of interest to the loading control as follow **D.** Cleaved PARP/ β-Actin **E.** Cleaved Caspase-3/β-Actin. **F.** Protein expression in figure 5-D has been quantified as a ratio between TRPV2 to the loading control GAPDH in non-targeting small interfering RNA (SUM-NT SiRNA) and TRPV2-targeting small interfering RNA (SUM-TRPV2-SiRNA) transiently transfected cells. Protein expressions in figure 6-D have been quantified as a ratio between the protein of interest to the loading control as follow **G.** Cleaved PARP/ β-Actin **H.** Cleaved Caspase-3/ β-Actin **I.** TRPV2/GAPDH.

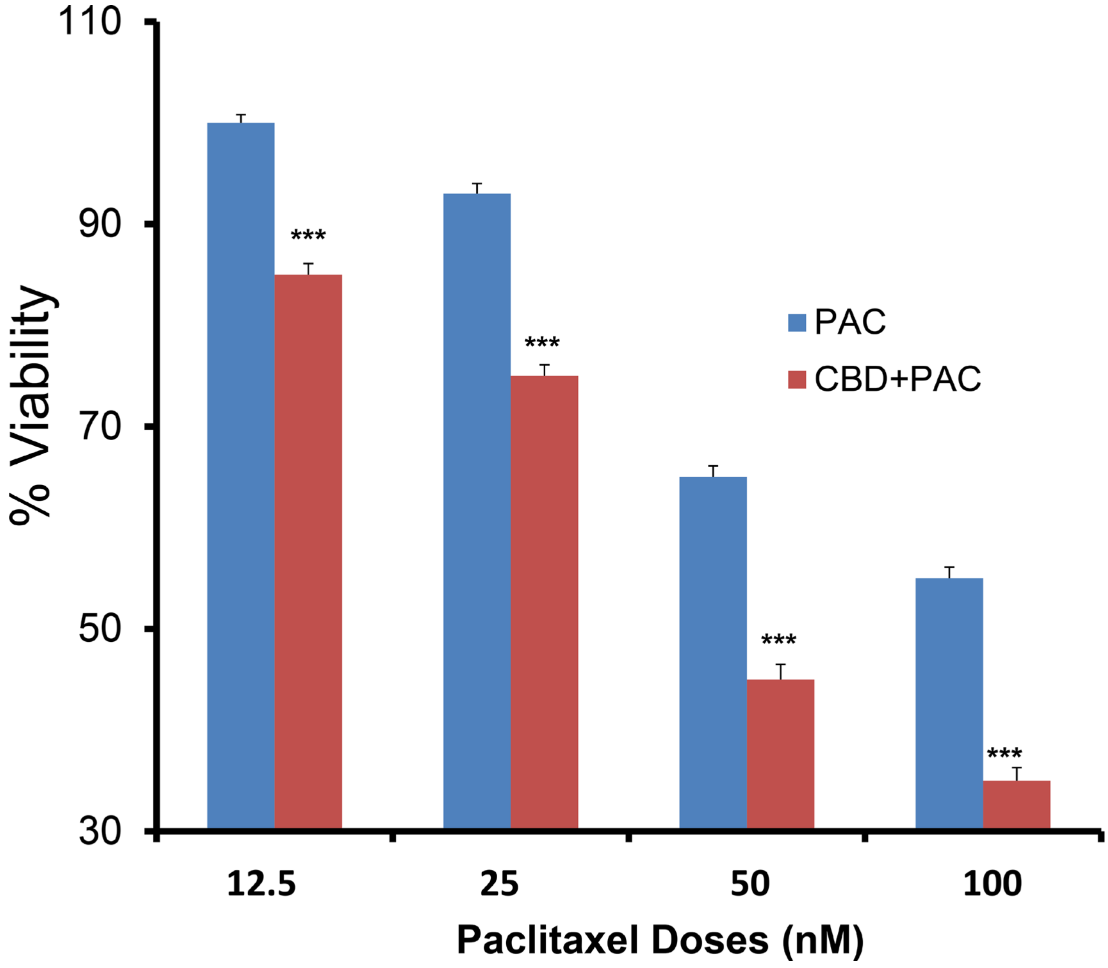

Supplementary Figure S2: CBD potentiates Paclitaxel's efficacy. SUM159 cells were treated with different concentrations of Paclitaxel (Pac) in presence or absence of CBD for 24 hours and subjected to MTT assay.
